# The epidemiology, radiology and biological characteristics of interval breast cancers in population mammography screening

**DOI:** 10.1038/s41523-017-0014-x

**Published:** 2017-04-13

**Authors:** Nehmat Houssami, Kylie Hunter

**Affiliations:** 1 Sydney School of Public Health, Sydney Medical School, University of Sydney, Sydney, NSW Australia; 2 National Health and Medical Research Council (NHMRC) Clinical Trials Centre, Sydney Medical School, University of Sydney, Sydney, NSW Australia

## Abstract

An *interval* breast cancer is a cancer that emerges following a negative mammographic screen. This overview describes the epidemiology, and the radiological and biological characteristics of interval breast cancers in population mammography screening. Notwithstanding possible differences in ascertainment of interval breast cancers, there was broad variability in reported interval breast cancer rates (range 7.0 to 49.3 per 10,000 screens) reflecting heterogeneity in underlying breast cancer rates, screening rounds (initial or repeat screens), and the length and phase of the inter-screening interval. The majority of studies (based on biennial screening) reported interval breast cancer rates in the range of 8.4 to 21.1 per 10,000 screens spanning the two-year interval with the larger proportion occurring in the second year. Despite methodological limitations inherent in radiological surveillance (retrospective mammographic review) of interval breast cancers, this form of surveillance consistently reveals that the majority of interval cancers represent either true interval or occult cancers that were not visible on the index mammographic screen; approximately 20–25% of interval breast cancers are classified as having been missed (false-negatives). The biological characteristics of interval breast cancers show that they have relatively worse tumour prognostic characteristics and biomarker profile, and also survival outcomes, than screen-detected breast cancers; however, they have similar characteristics and prognosis as breast cancers occurring in non-screened women. There was limited evidence on the effect on interval breast cancer frequency and outcomes following transition from film to digital mammography screening.

## Introduction

### Aims

A breast cancer (BC) that emerges following a negative mammographic screen is referred to as an interval BC.^1^ In this overview, we describe the epidemiology, radiology and biological characteristics of interval BCs in population mammography screening, highlighting published research from the most recent decade. The aims of the review were to provide an update on interval BCs that extends both our work on radiological surveillance of interval BCs^1^ and that from other researchers that have quantified interval BC rates,^2–4^ to elucidate evidence on interval BCs following transition to digital mammography screening, and to identify knowledge gaps that warrant further research.

### Background and definitions

**Fig. 1:**
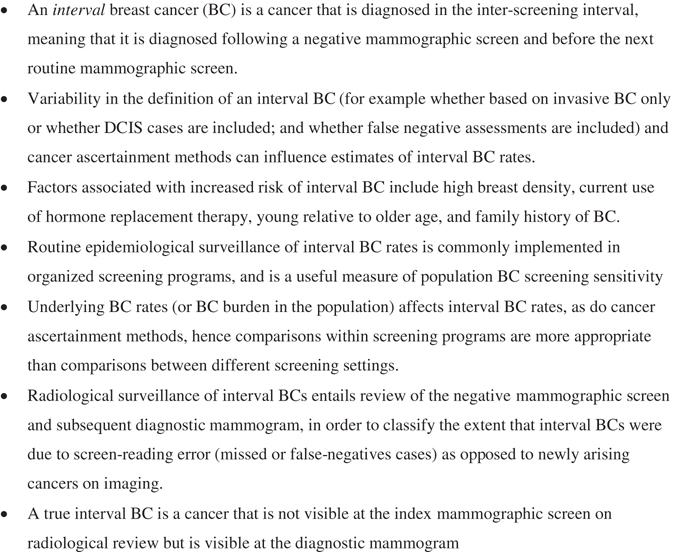
Summary of background definitions and themes

An interval BC refers to a cancer that presents after a ‘normal’ screening mammogram and before the next scheduled mammogram, in other words a BC that arises or is diagnosed in the inter-screening interval [see also Fig. 1].^1^ This definition may be qualified by specifying an interval case as an invasive BC,^5^ given that the vast majority of interval cases are invasive malignancies and much of the routinely reported data on interval BC rates is based on invasive BC. In addition, some qualify the definition further by specifying that interval BCs are those that arise clinically^2, 5^ in the inter-screening interval—although that would be the likely presentation for almost all interval BCs it should be noted that a BC identified in the inter-screening interval would still be classified as such irrespective of how it came to be diagnosed. Factors that have been associated with increased risk of an interval BC in screened women include high mammographic breast density,^6–8^ current use of hormone replacement therapy,^8, 9^ young relative to older age (partly reflecting confounding from breast density), however, absolute incidence rates are higher in older women given higher underlying BC rates,^8^ previous false-positive mammography,^10^ and a family history of BC.^8, 10, 11^

Because interval BCs are representative of the sensitivity of population breast screening, and given that they are an adverse outcome for women partaking in screening, surveillance of interval BCs is routinely practiced in many screening programs. Surveillance comprises epidemiologic measures (such as interval BC rates)^1, 3^ to monitor the frequency of interval cases, and may be complemented by radiological surveillance as part of quality assurance in organised screening programs.^1^ Various methodological and analytic parameters can substantially influence estimates of interval BC rates and other epidemiologic measures of interval BCs, as highlighted by several investigators.^3, 12^ These include variability in the definition of an interval BC (whether based on invasive BC or whether ductal carcinoma in-situ (DCIS) cases are included); whether false-negative assessment cases and lapsed attenders are counted or excluded; the adequacy of ascertainment of interval cancers, hence also the adequacy of cancer notification and registration; and the duration of follow-up for ascertainment of cases.^3, 12^ Importantly, underlying BC rates or burden in the population also affects interval BC rates. For these reasons, epidemiologic measures of interval BCs are best suited for monitoring within screening services or programs because comparisons between screening programs and countries is limited by heterogeneity in the above-described variables that affect quantitative estimates of interval BCs.

Radiological surveillance is a qualitative form of surveillance that defines and measures the extent that interval BCs represent screen-reading ‘errors’ as opposed to being non-detectable cancers at mammography screening. Radiological surveillance entails review of the mammograms taken at the time the interval BC is identified (usually at clinical presentation, hence the diagnostic mammograms) and the pre-diagnosis mammographic screen (the ‘negative’ index screen) and an interpretative judgement to classify each case into pre-defined categories.^1, 13–15^ These categories may vary in definition, however, most include a ‘true interval’ category (where the cancer is not visible at the index screen but becomes visible at the diagnostic mammogram) and a ‘missed’ interval BC being the equivalent of a false-negative (where the cancer is visible on the index mammogram but is not recalled or is misinterpreted) and is at times referred to as screening error.^1, 13–15^ Various methods have been used to perform radiological surveillance, as described in a review by Houssami et al.^1^ with potential biases inherent in the review strategy and the extent that readers are informed that they are evaluating interval BC cases.^1^ Notwithstanding the methodological limitations of radiological surveillance, it provides insights into screening quality and on how screening could be improved.

In the present review, we consider both epidemiological and radiological aspects of interval BCs, and complement these with information on tumour prognostic characteristics of interval BCs, to define common themes as well as evidence gaps, to enhance our understanding of interval BCs and inform research priorities.

## Results

### Epidemiologic surveillance

Table 1 presents a summary of epidemiologic measures for interval cancers including interval BC rates, which were the most commonly reported estimates for routine screening monitoring.^4, 13, 15–29^ The table highlights the broad variability in both interval BC rates and cancer detection rates at screening, both of which are partly driven by underlying cancer rates in the populations reported in these studies. There is wide variability in the *overall* interval BC rates, ranging between 7.0 and 49.3 per 10,000 screens, partly explained by data shown for screening rounds (initial and repeat screens) and the duration and year of the inter-screening intervals; where reported, data for the inter-screening interval are presented by yearly rates for biennial or triennial screening. If restricted to studies of annual screening or to year 1 data from biennial screening programs, there is evidence that interval BC rates are consistently <8/10,000 screens. The majority of studies in Table 1 report data for biennial screening programs: the interval BC rates spanning the two years between screens are within the range of 8.4 to 21.1 per 10,000 screens, with the larger proportion of the estimated interval BC rates occurring in the second year of a two-yearly interval. The evidence also consistently shows that interval BC rates are higher at repeat (incident) rounds than initial (prevalent) screening rounds.

**Table 1:** Epidemiological measures of the frequency of interval breast cancers in population mammography screening

Study first author	Screening setting^a^; age-group screened	Cancer detection rate per 10,000 screens	Interval cancer rate per 10,000 screens reported for all screens: overall or by inter-screen interval	Interval cancer rate per 10,000 screens where reported separately for initial and repeat screens	Proportional interval cancer rate^b^	Percentage of cancers (from all BCs in screened women) that are interval cancers^c^
Weber^13^	Southern screening region of Dutch program (2000–11), 302,699 film-screen and 115,047 digital mammograms; 50–75 years	Digital 69 Film-screen 52	Digital 17 Film-screen 20	–	–	Digital 19.4% Film-screen 28%
O'Brien^4^	Irish population breast screening program (2000–07); 50–64 years	53.6 (initial 66.9; repeat 41.4)	Overall 19.2 year 1: 5.8 year 2: 13.4	Initial 19.6 Repeat 18.9	40%	26%
Henderson^21^	Breast Cancer Surveillance Consortium, USA (2003–11), 3,021,515 mammograms (40.3% digital, 59.7% film); 40–89 years	Digital 44.7 Film-screen 44.2	12-month interval: Digital 7.3 Film-screen 7.9	–	–	14.7% (annual screening)
Carbonaro^15^	Milan, Italy, population breast screening program (2001–06); 49–69 years	55.2	Overall 17.0	–	29.0% (initial 19%; repeat 39%)	23.3%
Renart-Vicens^26^	Girona, Spain, Health Region screening program (2000–06), 50–69 years	49.0	Overall 7.0	–	Range 9.3 to 47.7%	13%
Fong^18^	Breast Test Wales screening program (1998–2001); 50–64 years	51.0	Overall 34.8	–	–	38% (3-yearly screening)
Heidinger^20^	German mammography screening program/North Rhine-Westphalia cancer registry (2005–08), 50–69 years	[study of initial screening round] 81.0	Overall 23.2 Year 1: 7.4 Year 2: 15.7	(all data are for initial screens)	Year 1: 27% Year 2: 58%	22%
Bennett^16^	National Health Service breast screening programme England, Wales, and Northern Ireland (1997–2003); 50–64 years	60.4	Overall 29.1 year 1: 5.5 year 2: 11.3 year 3: 12.2	–	–	32.5% (3-yearly screening)
Tornberg^28^	Navarra, Spain, population screening (1990–96); 45–65 years	41.5 (initial 63.0; repeat 31.3)	Overall 8.4^d^ year 1: 2.1^d^ year 2: 6.3^d^	Initial 7.2^e^ Repeat 9.0^e^	25.9%^d^ (initial 22%; repeat 28%)^e^	17%^d^
Seigneurin^27^	Isere, France, population screening – time frames are for change from 1-view to 2-view mammography: (2002–04) 50–69 years; (1994–99) 50–69 years	2002–04: 70.4 1994–99: 53.0	2002–04: Overall 15.3 year 1: 3.2 1994–99: Overall 23.9 year 1: 6.8	2002–04: Initial 17.3 Repeat 13.6 1994–99: Initial 24.7 Repeat 23.2	2002–04: 31.2%^d^ 1994–99: 48.7%^d^	2002–04: 17.8% 1994–99: 31%
Bordas^17^	Norrbotten, Sweden, population screening program (1989–2002); 40–74 years	29.4	Overall 10.7 year 1: 5.1 year 2: 5.6	–	33.4%^d^	26.6%
Tornberg^28^	Florence, Italy, population screening program (1990–94); 50–69 years	77.1 (initial 91.0; repeat 40.1)	Overall 15.3 year 1: 3.9 year 2: 11.4	Initial 13.3^e^ Repeat 21.0^e^	34.4%^d^ (initial 30%; repeat 47%)^e^	17%^d^
Tornberg^28^	Turin, Italy, population screening program (1992–96); 50–59 years	78.7 (initial 86.1; repeat 62.6)	Overall 15.5 year 1: 5.5 year 2: 10.0	Initial 14.0^e^ Repeat 19.2^e^	35.0%^d^ (initial 35%; repeat 47%)^e^	16%^d^
Fracheboud^19^	Netherlands population screening program (1990–93); 50–69 years	57.3 (initial 65.7, repeat 34.6)	Overall 18.2^d^ year 1: 6.1^d^ year 2: 12.1^d^	Initial 18.1 Repeat 18.6	39.3%^d^	24%^d^
Törnberg^28^	Marseille, France, population screening (1993–98); 50–69 years	46.7 (initial 46.5; repeat 47.3)	Overall 17.4 year 1: 5.4 year 2: 12.1	Initial 17.3^e^ Repeat 18.1^e^	43.3%^d^ (initial 43%; repeat 45%)^e^	27%^d^
Törnberg^28^	Strasbourg, France, population screening (1989–97); 50–65 years	42.7 (initial 51.9; repeat 37.2)	Overall 21.3 year 1: 6.9 year 2: 14.4	Initial 20.5^e^ Repeat 22.0^e^	47.2%^d^ (initial 45%; repeat 49%)^e^	33%^d^
Törnberg^28^	Four counties, Norway (1996–97); 50–69 years	67.2	Overall 19.5 year 1: 4.5 year 2: 15.0	Initial 16.4^e^ Repeat n/a	48.7%^d^ (initial 41%; repeat n/a)^e^	22%^d^
Törnberg^28^	Pirkanmaa, Finland (1988–99); 50–69 years	36.3 (initial 41.6; repeat 32.3)	Overall 17.4 year 1: 6.5 year 2: 11.0	–	66.4%^d^	32%^d^
Lynge^24^	Copenhagen, Denmark (1991–93 [initial] and 1993–95 [repeat]); 50–69 years	(Initial 118.6; repeat 62.5)^b^	–	Initial 17.3^e^ Repeat 20.5^e^	(Initial 34%; repeat 40%)^e^	Initial 13% Repeat 25%
Njor^25^	Funen, Denmark (1993–95 [initial] and 1996–97 [repeat]); 50–69 years	(Initial 95.9; repeat 52.1)^b^	–	Initial 21.2^e^ Repeat 24.2^e^	(Initial 43%; repeat 47%)^e^	Initial 18% Repeat 32%
Törnberg^28^	Stockholm, Sweden, screening program (1989–97); 50–69 years	47.6 (initial 58.9; repeat 39.8)	Overall 21.1 year 1: 7.3 year 2: 13.8	Initial 20.4^e^ Repeat 21.7^e^	(Initial 40%; repeat 46%)^e^	Initial 26% Repeat 35%
Hofvind^22^	Norwegian breast cancer screening program (1996–2005 [initial] and 1988–2005 [repeat]); 50–69 years	56.4 (initial 64.8; repeat 49.2)	18.2	Initial 18.3 Repeat 18.2	51%^e^	Initial 22% Repeat 27%
Kellen^23^	Belgian province of Limburg population screening program (1996–2001); 50–69 years	101 (included prevalent screening)	Overall 49.3 (for 3-year interval)	–	Year 1: 21.7% Year 2: 11.3%	36.6% (biennial program but included 3-year interval BC data)
Vettorazzi^29^	Screening program in local health units of Veneto Region, Italy (1999–2002); 50–69 years	[initial screening round] 72.0^e^	–	(all data are for initial screens) 16.3^e^	29% (year 1: 21%;year 2: 46%)	18%

^a^ Except where specified as including data for digital mammography, data are for film-screen mammography. Screening is biennial unless otherwise specified

^b^ The ratio of the interval cancer rate to the expected underlying incidence rate (also referred to as proportionate incidence)

^c^ Percentage is a simple representation of the proportion of breast cancers occurring in screened women (counting both screen-detected and interval cases in the denominator) that are recorded as interval cancers, and can also be expressed as a ratio (also referred to as an 'interval cancer ratio'^2^)

^d^ Based on data from O'Brien et al.^4^

^e^ Based on data from Andersen et al.^2^

For the majority of studies (based on biennial screening) interval BCs represent around 17–30% of the cancers occurring in screening participants as summarised in the *simple proportion* in the last column of Table 1; that proportion is relatively lower for annual (14.7% based on one study) and higher for triennial (32–38%) screening intervals. The proportional interval BC rate, also shown in Table 1, is not reported by all studies because this measure requires estimating the expected underlying incidence in the absence of screening, so may not be feasible to calculate in contemporary screening practice.^2^

#### Mammography screening interventions associated with reduction in interval BC rates

A study conducted in the United Kingdom’s screening program, based on women aged 50–64 screened between 2003 and 2005, reported that two-view mammography (at the last routine screen) was associated with a reduction in interval BC rates of 6.8/10,000 screens compared with one-view mammography.^30^ The investigators concluded that this suggests that two-view (instead of one-view) mammography at incident screening was accompanied by a 15–20% reduction in interval BC rates. An earlier study by Seigneurin et al.^27^ reported similar evidence from the French population breast screening program, based on women aged 50–69, in a comparison of two time frames reflecting transition from one-view to two-view mammography: a reduction of 8.6 interval BCs per 10,000 screens was associated with two-view mammography, with an estimated 36% difference in relative risk of interval BC at 24 months for two vs. one view mammography (Table 1). Weber et al.^13^ reported a study of the Southern screening region of the Dutch program, in which the use of digital (compared to film-screen) mammography was shown to be associated with a modest but significant reduction in interval BC rates of 3/10,000 screens (*p* = 0.02; Table 1).

### Radiological surveillance

Contextual background for radiological review and classification of interval BCs, along with definitions of the categories of interval BCs, have been outlined in the introduction of the paper; methodological issues have been comprehensively explained in our previous review.^1^ Table 2 summarises findings from the literature search on radiological surveillance including the methods used to conduct mammographic review;^10, 13–15, 18, 26, 31–40^ the latter substantially influences the distribution of radiological categories and can bias estimated proportions.^1, 33, 41^ For example, a pilot study examining radiological review methods showed that informed vs. blinded (uninformed) review of interval BC leads to bias in classification whereby informed reviewers (aware they were reviewing mammograms containing interval cases) more frequently classified some interval BCs as positive, compared to reviewers who were unaware they were reading mammograms of interval BCs that had been added into routine screen-reading practice (‘uniformed’ review).^41^ To the extent that ‘blinding’ is possible in evaluation of interval BCs, this methodology sometimes referred to interval cases being interspersed with screen-reading as part of the routine screening workflow, or more frequently partial blinding was achieved by interval cases being mixed with normal screening mammograms (Table 2). Semi-informed radiological review methods involved knowledge that interval BC cases were being reviewed, without information on the side and location of the interval BC. In general, studies of radiological classification were based on an initial review of the index screen (the screen preceding the subsequently diagnosed interval BC) with provisional identification and classification of the interval BC, followed by review of both the screening and diagnostic mammograms to enable definitive classification or sub-classification. However, not all studies provided these details and some studies did not specify whether the diagnostic mammograms were available for classification. Interpretation was generally performed by experienced mammography readers, and varied from one expert screen-reader performing classification, to panels of several radiologists with classification based on reaching consensus or derived from majority reads. Table 2 footnote provides further definitions on radiological review methods and classification terminology.

**Table 2:** Radiological surveillance of interval breast cancers: methods and results of mammographic review and classification

Study (first author)	Methods	Distribution of radiological classification^a^ of interval breast cancers	Additional findings
Weber^13^	Review of 800 interval BCs from southern screening region of Dutch breast screening program (2000–11 spanning transition from FSM to DM) by two radiologists based on prior screen and diagnostic mammogram.	Year 1 of inter-screening interval True interval: 43.1% Missed/FN: 31.3% Minimal signs: 25.7% Year 2 of inter-screening interval True interval: 60.2% Missed/FN: 19.1% Minimal signs: 20.7%	Majority of missed or minimal-signs cases were masses at prior FSM or DM. No differences in mammography features (for FSM vs. DM) for cases emerging year 1 of inter-screen interval; however, cases in year 2 of inter-screen interval for DM were more frequently true (than missed) interval BCs compared to those for FSM (*p* = 0.03).
Blanch^10^	Review of 1012 interval cancers (Spanish screening program 2000–06) by panels of three experienced radiologists using semi-informed^b^ review of screening and diagnostic mammograms, independent double-reading and arbitration for discordant classification.	True interval: 48.2% Missed/FN: 23.2% Minimal signs: 17.2% Occult cancer: 11.3%	Factors associated with interval BC differed by radiological category, for example, family history of BC was mainly associated with true interval BC, whereas density was more strongly associated with occult BCs followed by true interval BCs.
Nederend^38^	Review of 224 interval cases from FSM or DM screening (prior screen and those taken at diagnosis) by two experienced radiologists: readers aware they were reviewing interval BCs but blinded to each other's review; consensus for discordant classification	True interval^c^: FSM 47.1%; DM 65.3% Missed/FN: FSM 30.8%; DM 20.2% Minimal signs: FSM 22.1%; DM 14.5%	Majority of missed interval BCs were masses at prior FSM or DM, followed by asymmetry or architectural distortion.
Domingo^14^	Study of 2245 invasive BCs (948 were interval cases) diagnosed 2000–09 in women participating in biennial population screening in Spain; interval BCs were classified by semi-informed^b^ review of the screening and diagnostic mammograms by panels of three radiologists.	True interval: 48.0% Missed /FN: 23.6% Minimal signs: 17.5% Occult cancer: 10.9%	True interval BCs were associated with HER2 and triple-negative tumour phenotypes and with extremely dense (>75% density) breasts; extreme breast density was most strongly associated with occult interval BCs
Renart-Vicens^26^	Review of 22 interval cases (Girona Health Region screening program 2000–06) by panel of expert radiologists, using semi-informed^b^ independent double-reads of screening and diagnostic mammograms, with arbitration for discordant classification.	True interval: 54.5% Missed /FN: 13.6% Minimal signs: 13.6% Occult cancer: 18.2%	Distribution of pathological features differed between interval and screen-detected BCs (see Table 3)
Fong^18^	Review of 692 interval BCs, with comparison to screen-detected BC (Breast Test Wales 1998–2001): blinded^d^ review of screening and 'symptomatic' mammograms by two readers, with consensus for discordant classification.	True interval: 57.8% Missed /FN: 17.7% Occult cancer: 10.0% Unclassified: 2.2%	10-year all-cause survival rate for screen-detected BC (81.6%) was higher than that for interval BC (72.4%) [*p* < 0.001]: this differed by radiological category, true interval BC (77.5%), FN interval BC (55%), occult (54.4%) with latter two types having lower survival rates than screen-detected.
Carbonaro^15^	Review of 130 interval BCs in population screening program, Italy 2001–06: three expert radiologists blindly reviewed mammograms, mixed with negative screens: cases not recalled classified as true interval BC, those recalled by only one reviewer as minimal signs, and those recalled by >2 reviewers as missed interval BCs	True interval: 55.0% Missed /FN: 22.0% Minimal signs: 24.0%	A higher rate of larger (T3-T4) tumours was evident for missed interval BCs (18%) than minimal signs (6%) or true interval BCs (8%); and the rate of node metastases (N2-N3) for minimal signs (19%) or missed cancers (25%) was higher than that for true interval BCs (10%).
Payne^39^	Review of 332 interval BCs (Nova Scotia screening program 1991–2004): blinded^d^ and independent review by three experienced radiologists; classified as true interval BC if >2 radiologists reported index screen as normal (otherwise classified as missed interval BC if >2 reported abnormal screen).	Classified into two categories: True interval: 74.1% Missed/FN: 25.9%	Breast density distribution varied between the two types of interval BC and differed across age-group; rate of true interval BCs was higher for longer screening interval but this was not the case for FN cases.
Pellegrini^40^	Review of 103 interval BCs in population screening program Trento, Italy 2001–08: external (three radiologists) and internal (five radiologists) panel with varying screening experience blindly reviewed pre-diagnosis screening mammograms, mixed with negative controls. Classification based on majority report ('missed' if recalled by most reviewers).	External review True interval^c^: 67.0% Missed/FN: 18.4% Minimal signs: 14.6% Internal review True interval^c^: 62.1% Missed/FN: 17.4% Minimal signs: 20.4%	No significant difference between external and internal radiological review.
Caumo^32^	Review of 100 interval BCs in Verona, Italy, screening program 2000–06: three expert radiologists blindly reviewed pre-diagnosis mammograms, mixed with negative controls. Classification according to majority report.	True interval^c^: 71.0% Missed/FN: 15.0% Minimal signs: 14.0%	Interval BC proportional incidence 10.8% in year 1 and 40.0% in year 2 of inter-screening interval. Interval BCs associated with denser breasts compared with negative controls (*p* = 0.02).
Pirola^34^	Review of pre-diagnosis screening mammograms of 30 interval BCs from Milan, Italy, screening program (2005) performed by an expert radiologist who had read >300,000 mammograms, blinded^d^ to interval BCs by case-mix with negative screens.	True interval^c^: 76.7% Missed /FN: 16.6% Minimal signs: 6.7%	Interval BC proportional incidence estimated as 17.4% for 2-year inter-screening interval.
Hofvind^37^	Review of 231 interval BCs in Norwegian population screening program 1995–98: six experienced radiologists reviewed and classified cases in a consensus meeting, using screening and diagnostic mammograms; classified as missed if all radiologists agreed tumour was visible at screening mammogram	True interval: 35% Missed /FN: 35% Minimal signs: 23% Occult cancer: 7%	Of the combined missed and minimal signs interval BCs, 50% were poorly defined masses or asymmetric densities, 26% were MC with/without associated density or mass, at the baseline screen.
Bare^31^	Review of 57 interval BCs in population screening program in Northeast Spain 1995–2001: 'informed consensus review' by three experienced radiologists using screening and diagnostic mammograms.	Excludes 19 'unclassifiable' cases: True interval: 39.5% Missed/FN: 21.1% Minimal signs: 26.3% Occult cancer: 13.2%	No major differences in the prognostic features of interval BCs when examined by radiological type or time elapsed since last screening mammogram.
Ciatto^33^	Independent review of 100 screening mammograms (20 interval BCs, 80 negative screens) by six radiologists, using three sequenced review methods (separated by 2 weeks) with increasing information: (1) blinded^d^ (no IC information, case-mix) (2) partially informed^b^ (aware IC) (3) fully informed (with diagnostic mammograms)	Method 1 average (range): Missed /FN: 24% (10–40) Minimal signs: 6% (5–15) Method 2 average (range) Missed/FN: 33% (20–55) Minimal signs: 10% (10–20) Method 3 average (range) Missed/FN: 42% (35–50) Minimal signs: 20% (15–30)	A classification of 'missed' or minimal-signs interval BC was more likely using method 2 (odds ratio (OR) = 1.78, *p* = 0.033 or method 3 (OR = 3.91, *p* = 0.000) relative to method 1, but no reader effect was evident.
Evans^35^	Review of 208 interval BCs from a multi-centre RCT of screening from age 40–41 years: review by two radiologists with arbitration by a third, using semi-informed^b^ review of screening mammograms followed by diagnostic mammograms. Abnormalities further classified as malignant, subtle (features difficult to detect), or non-specific (features only seen in retrospect after reviewing diagnostic films).	True interval: 42% Missed/ FN: 26% Occult cancer: 32%	Features frequently misinterpreted were granular MC (38%), asymmetric density (27%), distortion (22%). Of abnormal previous screens, 37% were classified malignant, 39% subtle change and 21% non-specific. MC more common on diagnostic mammograms of FNs than those of true interval BCs (28 vs. 14%). Cases with true interval or FN findings had similar background parenchymal patterns, but those with occult interval BC had higher proportion of dense patterns, *p* < 0.05
Gao^36^	Review of 59 interval BCs (Singapore screening program 1994–97) by three radiologists using index screens; semi-informed (aware reviewing interval cases but unaware of tumour location).	Missed/FN: 17% (based on 'worst diagnoses' from five screen-readers, two from initial reads and three from re-review).	In 3 years of successive follow-up from index screen, interval BC rates per 10,000 women-years were 2.1, 10.6 and 10.8 each year.

*BC* breast cancer, *DM* digital mammography, *FSM* film-screen mammography, *FN* false-negative, *MC* micro-calcifications, *IC* interval cancer, *RCT* randomised controlled trial

^a^ Classification of interval BCs: true interval (cancer is not visible at the index mammographic screen but becomes visible at the diagnostic mammogram); missed/FN (cancer is visible on the index mammogram but is not recalled or is misinterpreted); minimal-signs (subtle abnormality is visible on the index mammogram but one that is unlikely to warrant recall); occult (cancer that is not visible on the index screen and not visible on the diagnostic mammogram)

^b^ Semi-informed radiological review generally involved screen-readers knowing that interval BC cases were being reviewed, without information on the side and location of the interval cancer

^c^ In some studies 'true interval' BCs are also referred to as 'occult' at the index or pre-diagnosis screen; this should not be confused with the conventional 'occult cancer' classification of interval cases, which usually refers to a BC that is not seen on the index mammography screen and also occult on the diagnostic mammogram

^d^ Blinding or blinded methods of review: this generally refers to (a) interval cases being interspersed with screen-reading as part of the routine screening workflow; or (b) interval cases being mixed with normal screening mammograms but not integrated into routine screen-reading workflow (study-specific methods described in table)

As shown in Table 2, radiological categories varied slightly across studies, however, most studies reported the distribution for true interval BC and also for missed or false-negative cases (defined in the introduction); some studies reported the additional categories of ‘occult’ and ‘minimal-signs’ interval BCs (see Table 2 footnote). The evidence table shows that the vast majority of interval BCs were *not* missed at screen-reading but were true interval BCs (range 40% to 77%) or occult interval BCs (7% to 32%), meaning that they were not visible on the index screen even in hindsight. Of note, some of the high proportions reported for true interval BCs (>60%) appear to have included the occult cases among the true interval cases. The proportion of missed (false-negative) interval BCs ranged between 13.6% and 35%, with the majority reporting a frequency of 20–25% based on radiological surveillance. A study from Ciatto and colleagues^33^ used a multi-methods evaluation of the same set of mammograms, and showed that increasing the information available to screen-readers significantly increased the proportion of interval BCs classified as missed (Table 2), highlighting the impact of review methods on radiological classification.

Additional findings summarised in Table 2 describe radiological findings (where present) for the index screen, which were frequently masses or asymmetric densities. They also highlight study-specific data showing differences in the variables associated with interval BCs, and in the tumour characteristics of interval BCs, across radiological categories derived from mammographic review.

Radiological review of interval BCs following screening with digital or film-screen mammography was reported by Knox et al.^42^ showing that a similar proportion of cancers were classified as missed cases at digital and film-screen (10.5% and 8.1%, respectively, *p* = 0.77). However, fewer interval BCs were depicted as microcalcifications alone or in association with another imaging abnormality following digital than film-screen mammography (16% and 32%, respectively, *p* = 0.02) (ref. 42). Nederend and colleagues^38^ investigated interval BCs in a population-based study of regional screening units in the Netherlands, and showed that significantly more interval BCs were classified as true-negative or true interval cases (not visible at the index screen) at radiological review of prior digital than prior film-screen mammography (65.3% vs. 47.1%, *p* = 0.02) as shown in Table 2; otherwise, there were no differences between interval BCs at digital or film-screen in terms of mammographic abnormalities at the prior screen or in tumour characteristics. Generally similar findings were reported by Weber et al.^13^ also from the Dutch screening program, who additionally observed that interval BCs emerging in year 2 of the inter-screen interval for digital mammography were more frequently true (than missed) interval cancers compared to those for film-screen mammography (*p* = 0.03; Table 2).

### Biological characteristics and prognosis

Table 3 summarises biological findings including tumour characteristics and biomarker profile for interval BCs, and outlines the comparison reported in each study because that accounts for some of the apparent heterogeneity in results.^11, 13, 14, 26, 31, 35–37, 39, 43–48^ For the majority of studies, which compared interval BCs with screen-detected cancers, there were consistent findings that interval BCs had worse prognostic features, such as larger tumour size, higher frequency of node metastases, higher histologic grade, and more advanced disease compared to screen-detected BC (Table 3). Although biomarker data were not consistently reported in these studies, where reported there was evidence that interval BCs had a higher frequency of triple-negative or HER2-positive cancers and a lower frequency of hormone receptor-positive cancers than screen-detected BC (Table 3).

**Table 3:** Biological characteristics of interval breast cancers

Study (first author)	Comparison and setting	Tumour characteristics and prognostic features (size, histology, grade, node status and/or stage)	Tumour biomarkers or phenotype-specific findings
Weber^13^	Interval vs. screen-detected BCs, southern region of Dutch screening program (2000–11); also compares interval BCs by radiological category and by year 1 vs. year 2 of inter-screen interval.	Interval BCs had higher proportions of T2+ tumours (52% vs. 21.5%) and of metastatic nodes (46.3% vs. 7.7%) than screen-detected BCs; interval BCs had different tumour histology distribution (fewer in-situ, higher proportion of invasive lobular) to screen-detected BCs. Missed cases had larger mean invasive tumour size than true intervals (28.5 vs. 24 mm, *p* = 0.003).	Interval BCs in year 2 of inter-screen interval for digital mammography were more frequently receptor triple negative than those occurring year 2 following film-screen mammography (*p* = 0.02).
Meshkat^47^	Interval vs. screen-detected BCs, screening unit for the Irish breast screening program (2010–13)	Interval BCs had higher tumour grade (*p* < 0.05) and higher stage (proportion stage 1 vs. 2; *p* < 0.001) than screen-detected BCs. Invasive lobular was more frequent among interval than screen-detected BCs (21% vs. 11%, *p* < 0.05).	Interval BCs less likely to be ER positive (76% vs. 81%, *p* < 0.05) and more likely to overexpress HER2 (20% vs. 10%, *p* < 0.05) than screen-detected BCs.
Holm^45^	Interval vs. screen-detected BC among women diagnosed with invasive BC (2001–08), Stockholm, Sweden, by breast density.	Interval BCs in non-dense breasts (<20% density) were more likely to harbour lymph node metastases (OR 3.55) than screen-detected BC in non-dense breasts.	Interval BCs in non-dense breasts more likely to be ER negative (OR 4.05), PR negative (OR 2.63), HER2 positive (OR 5.17), and triple negative (OR 5.33) than screen-detected BC.
Domingo^14^	Study of 2245 invasive BCs (948 were interval BCs) diagnosed 2000–09 in participants in population breast screening in Spain: compares interval and screen-detected BC, as well as categories of interval BCs, by density	Mean tumour size significantly larger for all radiological categories of interval BCs (range from 19.3 mm for occult cases to 25.3 mm for true interval cases) than mean tumour size for screen-detected BC (15.7 mm) [*p* < 0.001 comparison across all groups]. Proportion with lymph node metastases higher for all categories of interval BCs (range from 38% for occult cases to 50% for true interval and minimal-sign cases) than screen-detected BC (30%), [*p* < 0.001 comparing all groups]. Proportion with grade III tumours higher for all categories of interval BCs (range from 24% for occult to 45% for true interval cases) than screen-detected BC (21.6%), [*p* < 0.001 comparing all groups].	True interval BCs were associated with HER2 and triple-negative phenotypes (OR = 1.91 and OR = 2.07, respectively) and extremely dense breasts (OR = 1.67). Among true interval BCs, triple-negative tumours were more frequently observed in fatty (<25% density) than in denser breasts (*p*<0.001). FNs and occult interval BCs had similar phenotypic characteristics to screen-detected cancers.
Renart-Vicens^26^	Interval vs. screen-detected BCs from Girona Health Region screening program 2000–06.	Interval BCs had significantly higher proportions of advanced stage disease (14% vs. 1%), larger tumours (5.4% vs. 2.3%), high-grade tumours (38% vs. 23%), and higher number of metastatic nodes (13.5% vs. 7.7%) than screen-detected BCs.	Interval BCs were non-significantly more likely to be triple-negative, and less likely to be luminal A tumours than screen-detected BCs.
Boyd^11^	Interval vs. screen-detected BCs sourced from three case–control studies nested in screened populations, by density measured with various methods.	Interval BCs had significantly larger (average) maximum tumour diameter for each measure of density (percent mammographic density, dense and non-dense areas) than screen-detected BCs.	–
Caldarella^43^	Interval vs. screen-detected BCs, Florence population screening program 2004–05.	Stage at diagnosis was more advanced for interval BCs than screen-detected BCs based on pT distribution (pT 2+ 25.8% vs. 10.4%, *p* < 0.001) and pN distribution (pN 1+ 41% vs. 29%, *p* = 0.032).	Relative to screen-detected BC, triple-negative BCs were over-represented, and luminal A (ER/PR positive, HER2 negative) BCs were under-represented among interval BCs
Payne^39^	Interval vs. screen-detected BCs from Nova Scotia screening program 1991–2004.	Interval BCs were more likely to be node-positive, to be larger tumours, to have higher grade, and to show lymphovascular invasion than screen-detected BCs (all *p* < 0.001).	Interval BCs less likely to be ER positive than screen-detected BCs (*p* = 0.002).
Kalager^46^	Interval BC in the Norwegian screening program vs. BCs in same time frame in population not yet invited to screening (non-screened women).	Interval BCs had slightly higher proportions of larger tumours (>20 mm), stage II rather than stage I cancer, invasive lobular histology, and negative (non-metastatic) axillary nodes, than BCs in non-screened women (distributions for these variables differed at *p* < 0.001).	–
Caumo^44^	Interval vs. screen-detected vs. clinical BCs occurring in the absence of screening, Verona mammography screening program 2000–06 and Veneto cancer registry.	Interval BCs had more aggressive features than screen-detected BCs for pT (*p* < 0.001), pN (*p* < 0.001), and tumour grade distributions (*p* = 0.007). Interval BCs had similar prognostic features as clinical BCs based on pT, pN and grade distributions (all *p* > 0.05).	Interval BCs less likely to be ER-positive (77% vs. 91%, *p* < 0.001) and PR-positive (61% vs. 82%, *p* < 0.001) than screen-detected BCs. Interval BCs had similar proportions of ER/PR receptor positivity as clinical BCs.
Hofvind^37^	Comparison of interval BC subgroups (missed vs. minimal signs vs. true and occult) from Norwegian screening program, 1995–98.	Missed interval BCs had generally less favourable characteristics than true (including occult) interval BCs: average invasive cancer size 23 mm in missed vs. 18 mm in true interval BCs (*p* = 0.017). Higher proportion of interval BCs with node metastases among missed (49%) and minimal-signs (53%) than true interval (33%) BCs. Histological type did not differ between interval BC subgroups, but invasive lobular was more frequent in the missed (20%) than true interval BCs (9%) *p* = 0.06	ER and PR receptor status distribution did not differ between subgroups of interval BC.
Bare^31^	Comparison of interval BC subgroups (true + occult vs. minimal signs vs. FN vs. unclassifiable) from Northeast Spain screening program, 1995–2001.	No significant differences between the different radiological types in stage, tumour size, node status, histological grade, nuclear grade or histology. Minimal-signs group more frequently had poor prognosis based on NPI, whereas most frequent NPI classification for other groups was moderate (*p* = 0.003). Higher frequency of invasive lobular BC among false-negative BCs.	No significant differences between groups in ER or PR status.
Evans^35^	Comparison of interval BC type (true vs. FN vs. occult) from a multi-centre randomised trial, UK, conducted in younger age group (40–48 years).	Occult interval BCs were more likely to be <10 mm and <15 mm in invasive size than other interval BCs (*p* = 0.03 and 0.005, respectively). True interval BCs were more likely to be histologically grade 3 than other cases (*p* = 0.04). No evidence of an excess of lobular BCs in occult group.	–
Porter^48^	Comparison of 538 interval BCs by radiological type, in a UK screening program service, 1987–2000.	True and occult interval BCs (combined) were more likely to be histological grade 3 than minimal-signs and FN cases (52% vs. 35%, *p* = 0.05). FNs were more likely to have lobular histology than other interval BCs (47% vs. 20%, *p* < 0.001).	–
Gao^36^	Interval vs. screen-detected BCs from Singapore screening program 1994–97; also reported comparison to those declining screening and those not invited to screening.	Interval BCs were more likely to be stage II (52.5% vs. 31.1%) and have a tumour size >20 mm than screen-detected BCs, but less likely to be DCIS (10% vs. 26.5%). Distribution of axillary nodal status was similar between screen-detected and interval BCs; however, proportion of high-grade tumours among interval BCs (38%) was higher than screen-detected BCs (18.6%) and was similar to non-screened groups.	–

*BC* breast cancer, *ER* estrogen receptor, *FN* false negative, *HER2* human epidermal growth factor receptor 2, *PR* progesterone receptor,*OR* odds ratio, *NPI* Nottingham prognostic index, *DCIS* ductal carcinoma in-situ

Some studies compared prognostic features between radiological categories of interval BCs, with variable findings (and limited statistical comparisons once study data were examined in subsets), however, some differences were noted between radiological subgroups. These differences by radiological subgroup are detailed in Table 3, and suggest that the ‘missed’ group had worse prognostic features than the true interval and occult interval cancers, except for tumour grade, which was reported to be more frequently higher for true interval and occult interval BCs than missed cases. Additional findings suggest that these outcomes may differ slightly between dense and non-dense breasts but density-specific findings were reported in very few studies (Table 3).

In the limited number of studies comparing interval BCs with clinically presenting cancers in non-screened women,^44, 46^ there was evidence that interval cancers were similar in terms of prognostic features to the BCs occurring in non-screened women, however, one study reported that interval BCs had slightly higher proportions of larger tumours (>20 mm) than BCs in non-screened women.^46^

#### Prognosis of interval BC

A population-based cohort study found similar survival for women who had an interval BC in the Norwegian screening program (hazard ratio (HR) 0.98; 95% confidence interval 0.84–1.15) as those who had BC diagnosed in the same time frame but had not yet been invited to mammography screening (non-screened women).^46^ A study from the Malmo mammography screening program^49^ showed that interval BCs from the first decade of service screening had similar stage distributions and survival as the BCs diagnosed in non-attenders to screening, whereas the screen-detected cancers in that time frame had more favourable stage distributions and survival than the interval cases. In this same study, there was also evidence that the prognosis of women with interval BC had improved over a 20-year period, as may be expected from overall improvements in BC prognosis over time.

Domingo and colleagues^14, 50^ conducted several studies examining the characteristics of interval BCs; one of these evaluated 2245 invasive BCs and clearly showed that interval BCs had more advanced tumours than screen-detected BCs (additional details by interval BC category shown in Table 3).^14^ In an earlier study of 228 invasive BCs diagnosed among Barcelona women aged 50–69 years, Domingo et al.^50^also found that disease-free survival rates (at 5 year follow-up from diagnosis) for screen-detected, true interval, and symptom-detected BC were 87.5%, 64.1%, and 79.4%, respectively, and overall survival rates were 94.5%, 65.5%, and 85.6%, respectively.^50^ In keeping with these findings, they concluded that clinically-detected BC especially where these are true interval cancers had worse prognosis and poorer survival than screen-detected BC even after adjustment for clinical-pathological variables.^50^ Porter and colleagues^48^ compared the features of interval BCs by radiological classification, and although they observed differences in the histological characteristics (shown in Table 3) there was no significant survival difference between interval BC radiological types (*p* = 0.64).

Some studies have examined the prognostic characteristics of interval BCs by breast tissue density.^11, 45^ Holm et al.^45^ showed that interval BCs occurring in non-dense breasts (defined by<20% density) had poorer prognostic features than screen-detected BC (Table 3), whereas interval BCs in dense breasts (≥50% density) were phenotypically more similar to screen-detected cancers. Eriksson et al.^51^ compared survival in interval and screen-detected BC allowing for mammographic density in women aged 50 years and older; they showed that hazard rates for BC-specific survival were significantly higher for interval than for screen-detected cancers, independent of density. In addition, interval BC in women with non-dense breasts had increased 5-year survival HR (2.43, *p* = 0.001) compared to screen-detected BC in non-dense breasts, but this was not the case in women with dense breasts, in whom a difference in survival was not statistically evident between interval and screen-detected BC (5-year survival HR 1.41, *p* = 0.49) after adjustment for cancer size.^51^

## Discussion

**Fig. 2:**
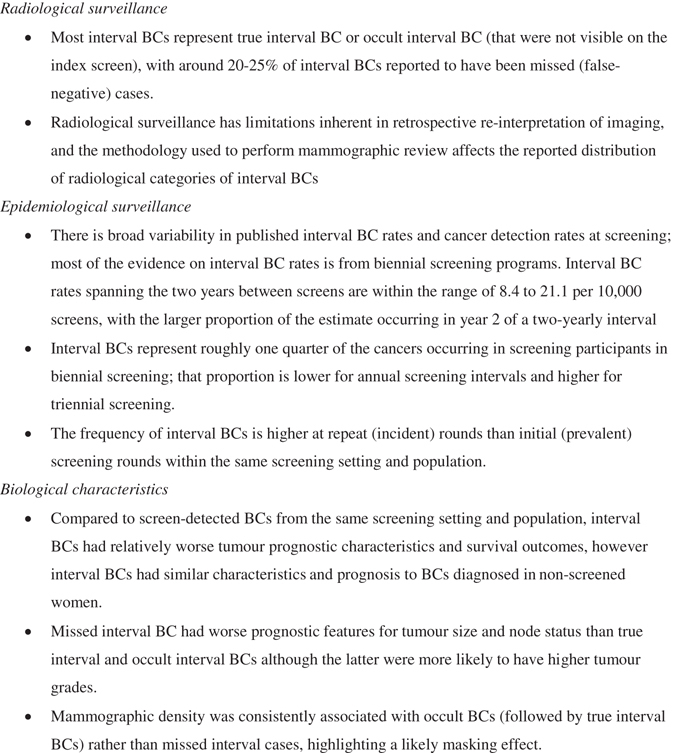
Key findings of the review

This overview of the epidemiological, radiological, and tumour characteristics of interval BCs—cancers that emerge following a negative mammographic screen—highlights key themes on interval BCs, which are summarised in Fig. 2. Interval BCs are an important consideration in population BC screening because they are indicative of screening quality hence evaluating these cancers may help identify areas for potential improvement, and because they represent a failure of screening to detect a BC that subsequently progresses to presentation. It is clear from radiological surveillance data summarised in our work that these cancers are not necessarily missed at mammography screening, with most studies reporting around 20–25% of interval BCs to be missed (false-negative) cases. While radiological surveillance has limitations inherent in retrospective re-interpretation of imaging, and radiological classification of interval BCs is affected by the methodology used to perform mammographic review (Table 2), quantitative evidence shows that the majority of interval cancers are true interval or occult interval BCs that were not visible on the index screen. It should be acknowledged that radiological surveillance is not practiced in all screening settings, and the aim of summarising the evidence is not to advocate this form of surveillance, rather its findings can inform practice. For example, it seems likely that enhancing screen-reader skills would have relatively less effect in controlling interval BCs than, say, enhancing mammography technology or using alternate or additional technology to address the majority of cases that are not visible at the index mammography screen even in hindsight.

Epidemiological monitoring of interval BC rates is a more widely performed surveillance in population mammography screening programs. Variability in the *overall* interval BC rates shown in Table 1 reflects the underlying BC risk in the population (which is also glimpsed in study-specific BC detection rates), the mix of initial and repeat screen rounds, and the length of the inter-screening interval. The variability due to the inter-screening interval is particularly evident where data are presented by yearly rates for biennial or triennial screening: our summary shows consistent evidence that rates in year 2 are at least twice those in year 1 (Table 1). The majority of studies in the evidence table report data from biennial screening, and show that interval BCs represent roughly one quarter of the cancers occurring in screening participants—that proportion is lower for annual, and higher for triennial, screening intervals. These findings should not be taken to infer that annual screening has better population outcomes than biennial screening (in fact biennial screening reduces some screening harms compared to annual screening), they merely highlight that many interval BCs are identified in the second year of a biennial screening round, and is commensurate with the pattern of findings from radiological surveillance specifically that many interval BCs are true interval cancers. Additional data from radiological surveillance from Weber and colleagues^13^ indicates that the proportion of interval cancers that are true interval BCs increases in year 2 (relative to year 1) at biennial screening. The evidence table also shows that interval BC rates are higher at repeat (incident) rounds than initial (prevalent) screening rounds within the same screening setting and population. This finding has not been thoroughly explained in the reviewed studies but is presumably due to age increase and a tendency for lower recall rates at repeat screening of the same women but warrants further research.

Although there is a substantial body of knowledge on interval BC rates in mammography screening, as shown in Table 1, there was little direct evidence on mammography-based interventions that reduce interval BC rates. A limited number of studies identified in this review reported that two-view (vs. one-view) mammography was associated with significant reductions in interval BC rates in population-based programs.^27, 30^ There was little evidence on the effect of digital mammography on interval BC rates, limited to one study showing that the use of digital (compared to film-screen) mammography was associated with a small but significant reduction in interval BC rates.^13^

Evidence on radiological features of interval BCs following transition to digital mammography was also limited to few studies^13, 38, 42^ with one study suggesting that implementation of digital mammography modified the mammographic pattern of interval BCs, with fewer interval BCs depicted as microcalcifications.^42^ However, two studies did not find substantial differences in interval BCs, in terms of the pattern of mammographic findings, following transition to digital mammography.^13, 38^

Studies providing data on the biological characteristics of interval BCs have mostly compared them to screen-detected BCs from the same screening setting and population, and have shown that interval BCs have relatively worse tumour prognostic characteristics, and worse survival outcomes, than screen-detected BCs. Interval BCs were consistently reported to be at a more advanced stage when diagnosed compared to screen-detected BC in terms of larger tumour size, higher frequency of node metastases, higher histologic grade, and had less favourable biomarker profile including a higher frequency of triple-negative cancers. This does not mean that interval BCs are an aggressive group of cancers, in fact they have tumour characteristics and survival outcomes that approximate those of BCs diagnosed in non-screened women, based on data from the few studies reporting that comparison.^44, 46^ One study reported that interval BCs had higher proportions of larger tumours than BCs in non-screened women but did not find any difference in survival between these groups.^46^ Further insights were provided by studies comparing prognostic features between radiological categories of interval BCs, with findings suggesting that the ‘missed’ group had worse prognostic features for tumour size and node status (possibly due to the delay in detection) than the true interval and occult interval BCs although the latter were more likely to have higher tumour grades (suggesting these to be more rapidly growing cancers representing new events on the mammogram).

Because mammographic breast density is an established risk factor for interval BC in screened women,^6, 7^ some of the studies summarised in this review evaluated interval BCs in relation to mammographic density.^10, 11, 14, 39, 45, 51^ The detailed findings, summarised in our results, are complex but reveal some common findings. Mammographic density was consistently associated with occult BCs (followed by true interval BCs) rather than missed interval cases,^10, 14^ highlighting a likely masking effect. In addition, there was a suggestion that interval BCs occurring in non-dense breasts may be associated with worse prognostic features and outcomes,^45, 51^ perhaps reflecting that interval BCs in non-dense breasts were more likely to be newly arising cases associated with rapid growth—however, the data were from few studies.

Epidemiological and radiological surveillance of interval BCs, complemented by an understanding of the biology of these cancers, provide insights into ‘how often’ and ‘why’ screening may not detect a BC that is subsequently diagnosed. Evidence shows that quantitative data on interval BCs are very heterogeneous and are influenced by several factors including the length of the inter-screening interval. Most published studies have reported data from biennial screening practice and in that context interval BCs represent roughly 17–30% of the cancers occurring in screening participants. Radiological surveillance highlights that the majority of interval BCs represent true interval or occult interval BCs that were not visible on the index mammographic screen, with only around 20–25% of interval BCs reported to have been missed cases on mammographic review. Biological characteristics of interval BCs show that they have relatively worse tumour prognostic characteristics and survival outcomes than screen-detected BCs, but similar characteristics and prognosis to BCs occurring in non-screened women. There was limited evidence of the effect on interval BC frequency and outcomes following transition from film to digital mammography screening.

## Methods

This is a descriptive review based on a literature search and extraction of relevant information into evidence tables for each of three themes: epidemiologic measures, radiological surveillance and biology of interval cancers in population mammography screening.

### Literature search

Relevant publications were identified through a Medline literature search: we exploded the term ‘breast neoplasms’ to August 2016, and combined this with title-searching for ‘interval cancer$’ or ‘interval breast cancer$’. Study identification focused on published work from 2006 onwards, given the above-stated aims of the review and the time frame from our previous evidence review,^1^ however, earlier studies were considered where data were reported in more recent publications.^2, 4^ Studies that provided information on population mammography screening allowing contribution into the evidence tables were included. Additional relevant studies were included in descriptive text if they provided key information on interval BCs that was not captured in the evidence table format. Studies that screened groups at increased risk of BC were not within the scope of the present review. Appendix 1 shows a flow diagram of the literature search and study inclusion process.

### Evidence tables

Each evidence table provided a summary of key findings from each study contributing information on interval BCs into at least one of three themes: epidemiologic measures, radiological surveillance and biologic features. For the evidence table on epidemiological measures (Table 1), studies were included if they reported data on interval BC rates (overall, or by year of inter-screen interval or by screening round) and also cancer detection rates because the latter provide complementary information about the study population and screening sensitivity. For the table on radiological surveillance (Table 2), studies were included if a radiological review and categorisation was performed allowing reporting of data on the frequency of one or more categories of interval BCs, and at minimum reporting data on false-negative (missed) interval cases. The evidence table on biological characteristics of interval BCs (Table 3) summarised data on tumour prognostic features, and also biomarkers where reported.

## Electronic supplementary material

Supplementary Information ‘Appendix 1: Flow-diagram of the literature search and study inclusion process’
