# Supplementary figures and images for: The epidemiology, radiology and biological characteristics of interval breast cancers in population mammography screening
Source: NPJ Breast Cancer. 2017 Apr 13;3:12. doi: 10.1038/s41523-017-0014-x (PMC5460204; doi:10.1038/s41523-017-0014-x)

## Appendix 1: Flow-diagram of the literature search and study inclusion process

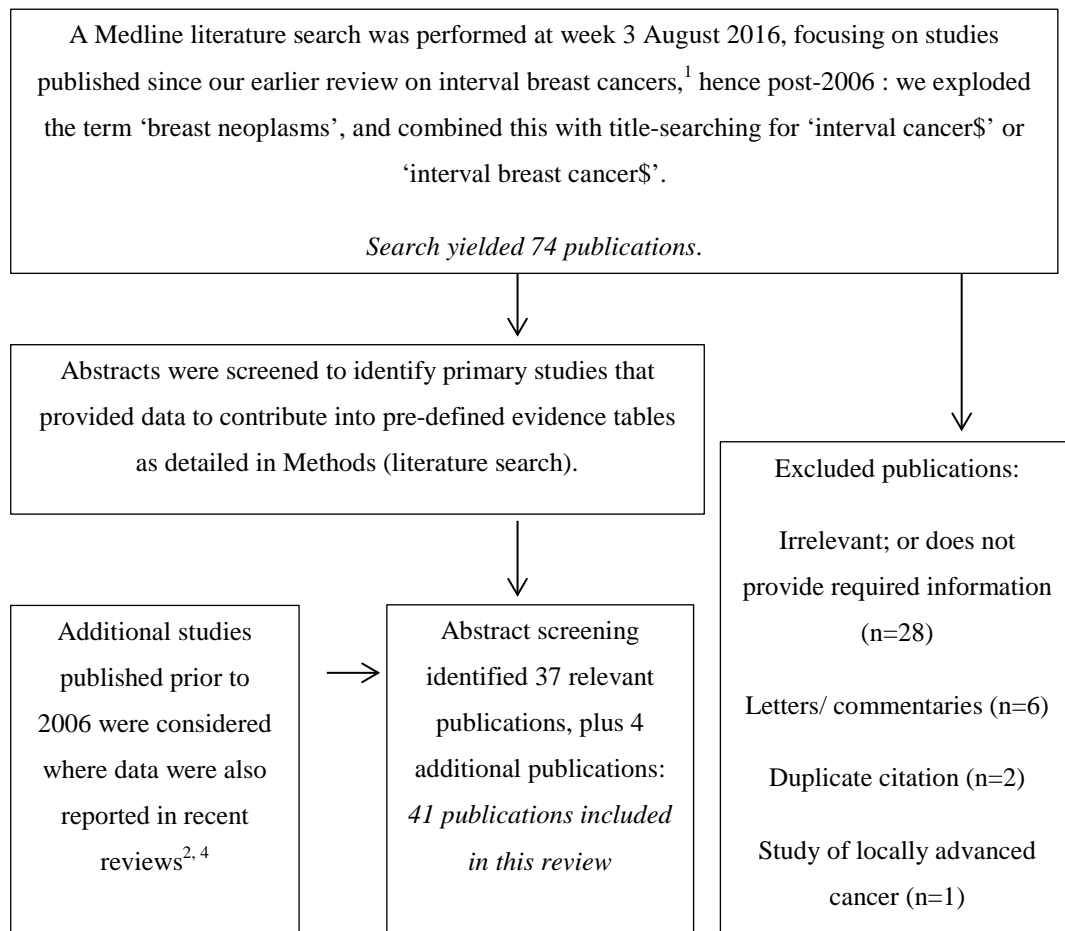

Supplement: Supplementary file 1 — Supplementary Information ‘Appendix 1: Flow-diagram of the literature search and study inclusion process’ [file 41523_2017_14_MOESM1_ESM.pdf]
